# Modulation of Oxidative Stress by Ozone Therapy in the Prevention and Treatment of Chemotherapy-Induced Toxicity: Review and Prospects

**DOI:** 10.3390/antiox8120588

**Published:** 2019-11-26

**Authors:** Bernardino Clavo, Francisco Rodríguez-Esparragón, Delvys Rodríguez-Abreu, Gregorio Martínez-Sánchez, Pedro Llontop, David Aguiar-Bujanda, Leandro Fernández-Pérez, Norberto Santana-Rodríguez

**Affiliations:** 1 Research Unit, Dr. Negrín University Hospital, 35019 Las Palmas de Gran Canaria, Spain; frodesp@gobiernodecanarias.org; 2 Chronic Pain Unit, Dr. Negrín University Hospital, 35019 Las Palmas de Gran Canaria, Spain; 3 Radiation Oncology Department, Dr. Negrín University Hospital, 35019 Las Palmas de Gran Canaria, Spain; 4 Universitary Institute for Research in Biomedicine and Health (IUIBS), Molecular and Translational Pharmacology Group, University of Las Palmas de Gran Canaria, 35016 Las Palmas de Gran Canaria, Spain; leandrofco.fernandez@ulpgc.es (L.F.-P.); norbesanrod@gmail.com (N.S.-R.); 5 Spanish Group of Clinical Research in Radiation Oncology (GICOR), 28290 Madrid, Spain; 6 Medical Oncology Department, Complejo Hospitalario Universitario Insular Materno-Infantil de Gran Canaria, University of Las Palmas de Gran Canaria, 35016 Las Palmas de Gran Canaria, Spain; delvysra@yahoo.com; 7 Scientific Advisor, Freelance, 60126 Ancona, Italy; gregorcuba@yahoo.it; 8 Experimental Medicine and Surgery Unit of Hospital Gregorio Marañon and the Health Research Institute of Hospital Gregorio Marañon (IiSGM), 28007 Madrid, Spain; perollonsa@gmail.com; 9 Medical Oncology Department, Dr. Negrín University Hospital, 35019 Las Palmas de Gran Canaria, Spain; dagubuj@gobiernodecanarias.org; 10 Thoracic Surgery, Department of Surgery, King Faisal Specialist Hospital and Research Center, 12713 Riyadh, Saudi Arabia; 11 College of Medicine, Department of Surgery, Alfaisal University, 11533 Riyadh, Saudi Arabia

**Keywords:** antioxidants, bleomycin, cancer treatment, chemotherapy-induced toxicity, cisplatin, doxorubicin, free radicals, methotrexate, oxidative stress, ozone therapy

## Abstract

(1) Background: Cancer is one of the leading causes of mortality worldwide. Radiotherapy and chemotherapy attempt to kill tumor cells by different mechanisms mediated by an intracellular increase of free radicals. However, free radicals can also increase in healthy cells and lead to oxidative stress, resulting in further damage to healthy tissues. Approaches to prevent or treat many of these side effects are limited. Ozone therapy can induce a controlled oxidative stress able to stimulate an adaptive antioxidant response in healthy tissue. This review describes the studies using ozone therapy to prevent and/or treat chemotherapy-induced toxicity, and how its effect is linked to a modification of free radicals and antioxidants. (2) Methods: This review encompasses a total of 13 peer-reviewed original articles (most of them with assessment of oxidative stress parameters) and some related works. It is mainly focused on four drugs: Cisplatin, Methotrexate, Doxorubicin, and Bleomycin. (3) Results: In experimental models and the few existing clinical studies, modulation of free radicals and antioxidants by ozone therapy was associated with decreased chemotherapy-induced toxicity. (4) Conclusions: The potential role of ozone therapy in the management of chemotherapy-induced toxicity merits further research. Randomized controlled trials are ongoing.

## 1. Introduction 

Chemotherapy (CT) is one of the main treatments for cancer. Its efficacy has been growing because of new chemotherapy agents, the new combination regimens and an increasing multimodal approach. Many effects of chemotherapy depend on the increase of free radicals and reactive oxygen species (ROS) in cancer cells. However, they can also mediate chemotherapy-induced toxicity (CIT). For many drugs, the most frequent and major toxicities are cytopenias, nausea, vomiting and hair loss. The latter is usually reversible; for the management and/or prevention of cytopenias, platelet or hemoglobin transfusions are available, as well as erythropoietin-stimulating agents and colony-stimulating growth factors (CSGF). However, some other major toxicities can affect different organs and tissues, depending on the CT agent. Usually, this damage is mediated by ROS and high oxidative stress, and frequently, preventive and therapeutic approaches are limited.

Cellular ROS are generated in mitochondria by oxidative phosphorylation. ROS also participate as signaling molecules in cell physiological processes of proliferation and survival. Thus, oxidative stress reflects the imbalance due to an excess of ROS or oxidants that overcome the capability of cells to exert effective antioxidant responses. Excessive ROS production may arise from mitochondria dysfunction or by the interaction between normal or excessive mitochondrial production with exogenous sources. The superoxide anion (O_2_•^−^) is a free radical produced by the single electron reduction of O_2_. It is the first ROS directly produced from O_2_ and the precursor of all other ROS. Spontaneous and superoxide dismutase (SOD)-dependent O_2_•^−^ dismutation generates hydrogen peroxide (H_2_O_2_), which itself can undergo the Fenton reaction to generate the hydroxyl radical (OH•) in the presence of transition metals, most commonly Fe^2+^. Oxidative stress results in macromolecular damage. Lipid peroxidation generates direct products such as malondialdehyde (MDA), isoprostanes, and 4-hydroxynonenal. Protein oxidation can cause fragmentation at amino acid residues, formation of protein-protein cross-linkages, and oxidation of the protein backbone. Oxidative damage to DNA causes alterations in DNA bases. Also, MDA can react with DNA to form DNA adducts [1,2].

Ozone (O_3_) is the triatomic allotrope form of oxygen which is much reactive (less stable) and more soluble (10 times) in water and plasma than the diatomic allotrope form (O_2_). Its antioxidant potency is the third after fluorine and persulfate and it is higher than O_2_ [3]. Ozone therapy consists in the medical use of a gas mixture of O_3_/O_2_, obtained from medical-grade oxygen using an ozone generator device and which has to be administered *in situ* because of the short half-life (at 20 °C the O_3_ concentration is halved within 40 min, at 30 °C within 25 min) [3]. Typical clinical O_3_ concentrations range from 10 to 60 µg/mL (µg of O_3_ / mL of O_2_) of a mixture O_3_ (0.5–0.05%) and O_2_ (95–99.5%) [4]. So, although more than 95% of the gas mixture is always oxygen, small variations in O_3_ content change its potential effects.

A higher concentration of ozone (maximum 0.02 µg/mL) is beneficial, preventing damaging UV light from reaching the Earth’s surface [5]. However, exposure by inhalation to prolonged ground-level ozone damages the respiratory system and extrapulmonary organs. In the same way, in humans, ozone can be dangerous or beneficial, depending on the route and organ/tissue of administration and on the concentration of exposition. It is becoming clear how the respiratory system—when undergoing a chronic oxidative stress—can release slowly, but steadily, a huge number of toxic compounds that are able to enter the circulation and cause serious damage [6]. Moreover, the potent antioxidant capacity of blood exposed to a small and precisely calculated dose of ozone only for a few minutes can modulate the endogenous antioxidant system and aids in the control of different pathological conditions [7].

This review is mainly focused on four drugs: Cisplatin, Methotrexate, Doxorubicin, and Bleomycin, which belong to different CT groups—alkylating agents, antimetabolites and antitumor antibiotics, respectively. These drugs can induce severe and dose-limiting toxicity, which has been reduced in experimental models when ozone has been administered as a preventive or therapeutic approach. Later, some related works supporting the previous studies will be summarized.

## 2. Chemotherapy-Induced Toxicity and Free Radicals

Mitochondria is one of the key contributors to cancer development and progression. Most of the O_2_•^−^ generated under physiological conditions are efficiently converted into H_2_O_2_ by superoxide dismutase (SOD). Catalase, glutathione peroxidase (GSH-Px, eight isoforms), and peroxiredoxins (Prxs, six isoforms) can convert H_2_O_2_ to water and O_2_, The O_2_•^−^ to H_2_O_2_ reaction also occurs spontaneously. Small ROS concentrations are required as messengers and signals for appropriated cell regulation. Higher levels of ROS and free radicals are produced by chemotherapy and radiotherapy as the main action mechanism for killing cancer cells. However, most of the chronic CIT are also influenced by the perpetuation of a pro-oxidative status and inflammation. Frequently, the most used approaches for many CIT include symptomatic treatments, substances with antioxidant effect and anti-inflammatory drugs and corticosteroids, although sometimes with limited efficacy. We include a short review of the action mechanisms and toxicities of the four drugs (Cisplatin, Methotrexate, Doxorubicin, and Bleomycin) that have undergone studies to evaluate CIT-modulation by ozone therapy.

### 2.1. Cisplatin-Induced Toxicity 

Cisplatin, cis- diamine-dichloro-platinum (CDDP) is one of the most used chemotherapy drugs because it is effective against different types of tumors. Cisplatin is an alkylating agent which is cell-cycle-phase nonspecific. It can bond to proteins, RNA and DNA, inhibiting DNA synthesis and cell cycle and it can also induce apoptosis. The most common cisplatin-induced toxicities are nausea, vomiting, myelosuppression, ion alterations, alopecia, sterility and others. However, among the most relevant and dose-limiting are ototoxicity, peripheral neuropathy and especially nephrotoxicity. Today, it is suggested that cisplatin-associated toxicities are mainly induced by free radicals’ production, which will result in oxidative organ injury. The evidence is growing over the protective effects of antioxidants on cisplatin-induced adverse reactions, especially, nephrotoxicity [8,9,10]. The main route for cisplatin elimination is via the kidneys, and around one out of three to four patients treated with full doses of cisplatin could develop renal dysfunction; the percentage could be higher than 50% in children. This damage can be produced at several renal structures: blood vessels (with vasoconstriction a decrease in renal blood flow), glomeruli and mainly, in proximal tubular cells [9,10].

### 2.2. Methotrexate-Induced Toxicity

Methotrexate (MTX) acts as an antimetabolite, blocking the dihydrofolate reductase and inhibiting the formation of tetrahydrofolic acid (reduced folic acid). This way, MTX inhibits formation of thymidylate from deoxyuridylate and inhibits the synthesis of DNA. This action and the additional inhibition of RNA and synthesis of proteins prevents cells to enter in the S phase of cell cycle (MTX is a cell cycle-specific agent).

MTX is used against many different tumors and in some autoimmune diseases such as rheumatoid arthritis. Although MTX is safely administered to most patients, it can cause significant toxicity, especially with chronic or high-dose schemes. In addition to myelosuppression, the most relevant could be pneumonitis (especially in irradiated areas), enteritis, leukoencephalopathy (intrathecal combined with high dose systemic administration) and especially, hepatic and acute kidney injury which can happen in 2–12% of patients. Nephrotoxicity results from crystallization of methotrexate in the renal tubular lumen, leading to tubular toxicity. Acute kidney injury and other toxicities of high-dose MTX can lead to significant morbidity, treatment delays, and diminished renal function [11]. The effects of MTX in vivo may be mediated by reducing cell proliferation, increasing the rate of apoptosis of T cells, increasing endogenous adenosine release, altering the expression of cellular adhesion molecules, influencing production of cytokines, humoral responses and bone formation. Several reports indicate that the effects of MTX are influenced by genetic variants, specific dynamic processes and micro-environmental elements such as nucleotide deprivation or glutathione levels [12]. MTX-induced toxicity has been related to oxidative stress [13] and down-regulation of the nuclear factor erythroid 2-related factor 2 (Nrf2) and heme oxygenase-1 (HO-1) [14].

### 2.3. Doxorubicin-Induced Toxicity

Doxorubicin (DOX) is an anthracycline antitumor antibiotic used against a large number of tumors. It is cell-cycle-phase nonspecific by intercalation between DNA base pairs, and it blocks the action of topoisomerase-II and inhibits the DNA and RNA synthesis. Similarly to many other chemotherapy agents, DOX frequently produces myelosuppression, nausea, vomiting, and alopecia. However, two potential DOX-induced toxicities are at cutaneous and cardiac level. DOX is a vesicant agent and its extravasation can produce local ulceration and necrosis. On the other hand, a potential and characteristic DOX-induced toxicity is cardiomyopathy with congestive heart failure. This cardiotoxic effect is dose-limiting and cumulative-dose dependent, with a high risk increase at cumulative doses higher than 550 mg/m^2^, or even lower (400 mg/m^2^) in patients with previous thoracic irradiation, previous cardiopathy or in combination with other drugs. Oxidative stress remains the most probable mechanism for the DOX-induced cardiotoxic effect, mediated by the production of iron-complex and the subsequent generation of free radicals [15,16]. In selected patients, Dexrazoxane can be used to prevent/diminish DOX-induced cardiotoxicity, because Dexrazoxane is an iron chelator that decreases the DOX-iron binding and the subsequent free radical generation.

### 2.4. Bleomycin-Induced Toxicity

Bleomycin (BLM) is a redox-active drug with anticancer and other clinical applications. BLM is an effective agent against lymphomas, testicular and ovarian germ cell cancers and certain squamous carcinomas. The antineoplastic effect of BLM is thought to involve the production of single- and double-strand breaks in DNA (scission) by a complex of BLM, ferrous ions, and molecular oxygen. Bleomycin binds to DNA by intercalation of the dithiazole moiety between base pairs of DNA and by electrostatic interactions of the terminal amines. The reduction of molecular oxygen by ferrous ions chelated by BLM leads to hydrogen subtraction from the C3 and C4 carbons of deoxyribose, resulting in cleavage of the C3–C4 bond and liberation of a base with a DNA strand break. BLM is inactivated in vivo by the enzyme BLM hydrolase, a cytosolic aminopeptidase that has lower activity in the skin and lungs. Bleomycin is selectively toxic to cells in the M and G2 phases of the cell cycle, and generally more effective against actively dividing rather than resting cells [17]. Despite being one of the most effective broad-spectrum chemotherapeutic agents in the treatment of cancers, the clinical applications of BLM have been limited due to the side effect of causing lung fibrosis [18]. The risk of BLM-induced fibrosis is increased by the improvements in overall survival and in those patients with previous lung diseases or thoracic irradiation.

The mechanism of BML-induced lung injury is not entirely clear, but likely includes components of oxidative damage, relative deficiency of the deactivating enzyme BML hydrolase, genetic susceptibility, and elaboration of inflammatory cytokines. Oxidative damage to the lung appears important in the pathophysiology of lung injury, and antioxidants may ameliorate the process [19]. Systemic administration of antioxidant artemisitene strongly inhibits bleomycin-induced lung damage, through the activation of the Nrf2 signaling pathway [20].

## 3. Modulation of Oxidative Stress by Ozone Therapy

Local ozone applications can induce direct effects and modulation effects at the local level. However, when ozone therapy is applied with systemic intent (principally by autohemotherapy and by rectal insufflation), ozone does not enter into the blood circulation and it is not able to reach any specific target tissues. Ozone that is not removed by the antioxidants of the medium interacts with unsaturated fatty acids from cell membranes in intestinal mucosa (rectal administration) or blood cells (in the extra-corporeal blood–ozone mixture, during auto-hemotherapy) generating aldehyde and hydroxy-hydroperoxide (ozone-peroxide), which forms H_2_O_2_ and a second aldehyde—4-hydroxynonenal (4-HNE), which is one of the most relevant aldehydes. These substances act as second messengers and induce a further adaptive response from the body (with potential over regulation of antioxidant systems) in a hormetic dose–response relationship [21,22,23]. This is, the action mechanism of systemic ozone therapy is an “indirect” effect. Ozone does not follow the standard principles of Pharmacology: absorption, distribution, metabolism and excretion. Ozone “only acts” as a modulator or pro-drug and, by inducing secondary messengers, will enhance the subsequent adaptive responses. After this fast reaction (few seconds), ozone disappears. Ozone concentration and effects do not follow a linear relationship: very low concentrations could have no effect and very high concentrations can lead to contrary effects to those produced by lower/middle concentrations [24]. Mediators such as 4-HNE and H_2_O_2_ are among the most relevant secondary messengers induced by ozone during lung toxicity following airway inhalation [25,26] but also, in the course of the induction of beneficial effects during medical application [2,27]. Moreover, H_2_O_2_ can enter the cytoplasm of mononuclear cells and modulate nuclear factor kappa B (NF-κB). H_2_O_2_ emerges not as an inducer of NF-κB, but as an agent able to modulate the activation of the NF-κB pathway by other agents. This modulation is generic at the level of the whole pathway but specific at the level of the single gene. Therefore, H_2_O_2_ is a fine-tuning regulator of NF-κB-dependent processes, as exemplified by its dual regulation of inflammation [28]. Most likely, the therapeutic dose of O_3_ blocks the NF-κB signal, reducing inflammation [29]. In contrast, a high dose of O_3_ promotes inflammation by activation of the NF-κB pathway [30]. In addition, H_2_O_2_ can act as promotor of the Nrf2 pathway. The important role of Nrf2 induction by ozone in order to enhance the antioxidant systems has been described recently [31,32,33].

There is a broad consensus on the relevance of the induction of protective molecules during small but repeated oxidative stress [22,34]. The most relevant aldehyde produced by the reaction of O_3_ is 4-HNE, which remains more stable than ROS [22,27]. 4-HNE is known to be quite reactive; it participates in multiple physiological processes as a nonclassical secondary messenger and readily forms covalent modifications of numerous targets [35]. 4-HNE is rapidly degraded by alcohol dehydrogenases, aldehyde dehydrogenase, and by glutathione-S-transferase. 4-HNE will form adducts with the thiol (-SH) and amino groups of Cys34 present in domain-I of albumin. This way, 4-HNE can send a signal of a transient oxidative stress to different tissues in the body and its effects depends on concentration as well as cell/tissue origin. This pathway can activate the synthesis of several substances such as: γ-glutamyl transferase, γ-glutamyl transpeptidase, HSP-70, HO-1, and antioxidant enzymes such as SOD, GSH-Px, catalase and glucose-6-phosphate dehydrogenase (G6PDH, a critical enzyme electron-donor during erythropoiesis in the bone marrow) and the Nrf2 pathway. In addition, these pluripotent effects of 4-HNE can be explained by its concentration-dependent interactions with the cytokine networks and complex cellular antioxidant systems also showing cell and tissue specificities [2,36]. As it happens with the potential actions of ozone, the potential actions of 4-HNE are very different at lower concentrations (regulation of proliferation and differentiation and enhancement of Nrf2 and antioxidant systems) than at high concentrations (induction of oxidative stress, apoptosis, and necrosis). 

**Figure 1:**
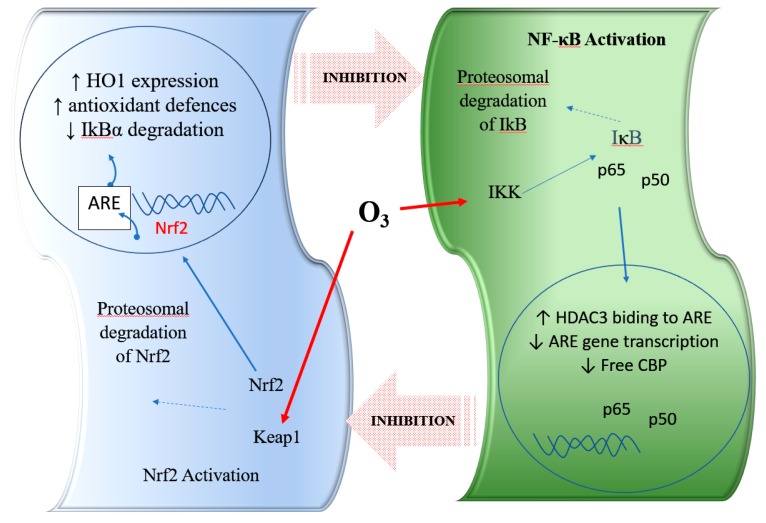
Representation of the interaction with the crosstalk between the nuclear factor erythroid 2-related factor 2 (Nrf2) and nuclear factor kappa B (NF-κB) pathways and the role of ozone. HO-1, haem-oxygenase-1; ARE, antioxidant response element; Keap1, Kelch-like ECH-associated protein 1; IKK: IκB kinase; CBP: CREB binding protein; HDAC3: histone deacetylase 3. Nrf2: nuclear erythroid 2 related factor 2; NF-κB: nuclear factor kappa light chain enhancer of B cell; LPS: lipopolysaccharide; O_3_: ozone.

Experimental results demonstrated that ozone ex vivo or in vivo can activate Nrf2 [7,37]. This mechanism can explain the genomic target of ozone, which induces the proteomic response (protein synthesis, as antioxidant enzymes: e.g., HO-1, SOD, CAT), providing far better protection against the total body damaging effects from free radicals. In addition, a very recent manuscript demonstrates the role of ozone on casein kinase 2 (CK2) (another regulator of the Nrf2 activity through its phosphorylation) in multiple sclerosis patients [38]. However, the effects of ozone also involve the modulation (inhibition) of the NF-κB pathway. This pathway activates the release of pro-inflammatory cytokines such as: TNFα, INFγ, IL1β, IL6, IL8, as well as pro-inflammatory genes such as cyclooxygenase-2 (COX-2) and inducible nitric oxide synthase (iNOS) [39]. As a result, the dose adminstered in ozone therapy and its hormetic response have a crucial role to manage the equilibrium inflammation/pro-inflammation responses. Both Nrf2 and NF-κB regulation are coordinated in order to maintain redox homeostasis in healthy cells. However, during pathological conditions, this regulation is perturbed, offering an opportunity for therapeutic intervention [39,40]. The regulation of inflammation by NF-κB signaling as well as Nrf2 pathways separately is widely documented. Since both these major signaling pathways modulate inflammation, they may crosstalk to bring about coordinated inflammatory responses (Figure 1) [41,42]. 

Preclinical studies indicated that ozone therapy could attenuate tubulointerstitial injury in rats with adenine-induced chronic kidney disease by mediating the modulation of Nrf2 and NF-κB [43]. In addition, clinical studies confirm this effect of O_3_ modulating the balance Nrf2/NF-κB in patients with multiple sclerosis [38].

## 4. Ozone Therapy in Chemotherapy-Induced Toxicity

Because ozone can modulate oxidative stress, inflammation and ischemia/hypoxia, it could be expected to exert a beneficial effect in chronic CIT when those mechanisms are involved. Several experimental models and isolated clinical studies have demonstrated its benefit in the prevention and/or treatment of CIT by some chemotherapy drugs, especially Cisplatin, Methotrexate, Doxorubicin, and Bleomycin. Finally, we will describe some related studies that offer additional support to the protective effect of ozone against CIT.

### 4.1. Ozone and Cisplatin-Induced Toxicity

In the last 15 years, several experimental models have described the effects and potential action mechanisms of ozone for prevention (by ozone preconditioning) or for treatment (stablished alterations) of renal damage by cisplatin. 

**Figure 2:**
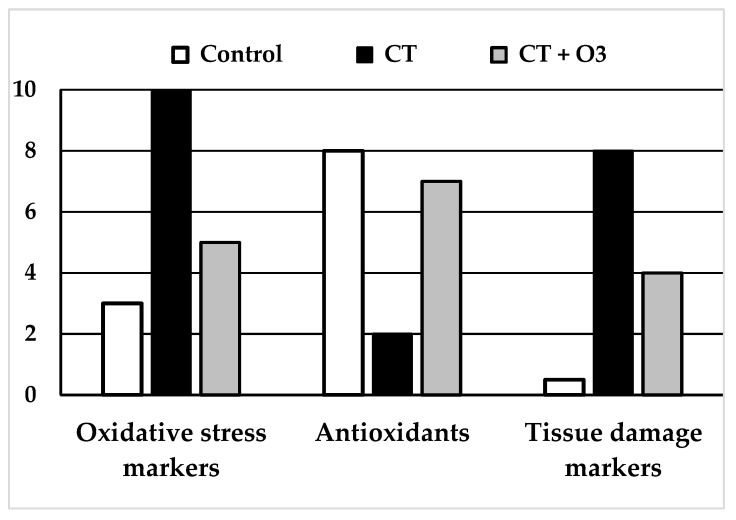
Schemes of results obtained in the experimental studies using systemic ozone therapy (rectal or intraperitoneal) using chemotherapy drugs. *(Left and Middle)*: “Oxidative stress markers” (MDA: malondialdehyde, TBARS: thiobarbituric acid-reactive substances) and “Tissue damage markers” (creatinine, pro-BNP: pro-brain natriuretic peptide) increased largely and significantly with chemotherapy. The increase was significantly lower in rats with chemotherapy + ozone therapy. *(Middle)*: levels of “Antioxidants” (GSH: glutathione, SOD: superoxide dismutase, CAT: catalase and GSH-GPx: glutathione peroxidase) decreased in chemotherapy group whereas those contents were closer to the control group in rats treated with chemotherapy + ozone therapy. All differences were statistically significant.

In 2004, Borrego et al. [44] described the effect of ozone preconditioning (ozone administration before cisplatin administration) to prevent cisplatin nephrotoxicity. Nine milliliters of ozone were administered, at different concentrations, by rectal insufflation: one session/day for 15 consecutive days before the day of intraperitoneal cisplatin injection. Rats were sacrificed 5 days after cisplatin injection. Regarding the control group without treatment, the groups with only O_2_ or with only O_3_ (without cisplatin) showed similar levels of serum creatinine (as a marker of renal damage), as well as renal levels of free radicals (measuring thiobarbituric acid-reactive substances—TBARS) and antioxidants (GSH, SOD, CAT, GSH-Px). Cisplatin group showed increased serum creatinine (four times) and TBARS (two times) and decrease of all antioxidants (between 15–40%). Regarding the cisplatin group, administration of cisplatin with O_2_ or with low O_3_ concentrations (10 µg/mL) did not show relevant changes and cisplatin with high O_3_ concentrations (50 or 70 µg/mL) showed similar (or even worse) creatinine levels, with disappointing results in antioxidants levels. Cisplatin plus O_3_/O_2_ preconditioning at these higher concentrations (50 and 70 µg/mL) showed histopathological changes that were quite similar to those present with cisplatin alone. However, rats treated with cisplatin and with O_3_ preconditioning at moderate concentrations (20 or 30 µg/mL) showed a relatively lower increase in creatinine levels (only two times) and TBARS, and a level of antioxidants similar or even higher than the levels of the control group. Patterns of change in levels of creatinine, free radicals and antioxidants were similar to those described in Figure 2. In the histopathological analysis, treatment with cisplatin alone showed intense tubular necrosis and cast formation in the lumen, whereas treatment with cisplatin O_3_/O_2_ preconditioning at 30 µg/mL showed no significant differences with non-treated rats.

Also in 2004, this group studied the effect of ozone administration after cisplatin-induced acute nephrotoxicity [45]. In this study, cisplatin was administered before the ozone treatment. After that, O_3_/O_2_ was administered at different concentrations (10, 30 and 50 µg/mL) by rectal insufflation: one session/day for five consecutive days. Rats were sacrificed one day later. Cisplatin alone or cisplatin + oxygen showed similar levels of all parameters, that is: the addition of oxygen had no effect. In comparison with the control group (without cisplatin), the cisplatin group showed significant increases in creatinine (marker of renal damage) and TBARS. All cisplatin + ozone groups showed levels of parameters closer to those of the control group, and a statistically significant difference with cisplatin alone: lower increase in creatinine and TBARS, and lower decrease (or even increase) of antioxidants. Additionally, treatment with cisplatin alone showed severe and widespread tubular necrosis with dilation of proximal tubules and cast formation in the lumen, whereas treatment with cisplatin and further O_3_/O_2_ also showed tubular necrosis, but to a lesser extent. Therefore, in the previous work, this group described a preventive effect against cisplatin-induced damage in the kidneys by ozone preconditioning [44], whereas the current study showed partial recovery of already established damage by ozone treatment after cisplatin administration [45]. Patterns of change in levels of creatinine, free radicals and antioxidants were similar to those described in Figure 2.

Later, in 2006 [46], the same group, evaluated the renal expression pattern of Bax in rats treated with cisplatin without/with O_3_/O_2_ administration only at the optimal O_3_/O_2_ concentration of 30 µg/mL, following the two previous approaches: (1) with the prevention approach of the 1st study, with ozone preconditioning administered before the injection of cisplatin (by rectal insufflation, one session/day during 15 days), and (2) with the treatment approach of the 2nd study, after cisplatin injection, (by O_3_/O_2_ rectal insufflations, one session/day during 5 days). Bax protein expression plays a relevant role in the induction of apoptosis. As described years before [47], cisplatin-induced toxicity was also associated with increased expression of Bax protein, in cytoplasm and nucleus in this work [46]. Overall, in the immunohistochemical analysis, rats receiving cisplatin injection and O_3_/O_2_ insufflations at 30 µg/mL showed lower expression of Bax, both in the preventive and treatment approaches, although the latter (with only five O_3_/O_2_ sessions) showed a smaller decrease in Bax expression, which was more relevant in the cortex zone. As in previous studies, compared with the control group, the increase in creatinine levels was significantly lower in rats treated with cisplatin and ozone, and an even better effect was demonstrated in the preventive group (15 days of O_3_/O_2_ insufflations) compared to the treatment group (with only five O_3_/O_2_ sessions).

It has been described that high levels of ROS can decrease the expression of Bcl-2 and increase the expression of Bax, with a final reduction of the ratio Bcl-2/Bax with a proapoptotic effect, as occurs with cisplatin-induced damage, whereas low doses of ROS can activate cell survival signaling pathways such as Nrf2 and its downstream HO-1, which can potentially decrease cytotoxicity [48]. In this way, HO-1 expression has been effectively described as a modulator of cisplatin-induced renal toxicity and its increase as a potential approach for decreasing kidney injury [49,50]. As described in these works, rectal O_3_/O_2_ insufflation at appropriated concentrations enhances the antioxidant mechanisms in renal tissue, which can explain its effect to prevent o diminish cisplatin-induced renal damage. Further support was provided years later, when it was described that appropriated O_3_/O_2_ concentration (this is, a moderate ROS stimulus) induces Nrf2 as the mechanism for increasing HO-1 [27] and antioxidant mechanisms leading to decrease in oxidative stress and pro-inflammatory cytokines [7,37,38,51]. 

Finally, in 2016, Kocak et al. [52] published a different experimental work, evaluating the effect of O_3_/O_2_ in the management of already established cisplatin-induced ototoxicity. Rats were treated with intratympanic and rectal ozone one session/days for 7 days. All rats received intraperitoneal cisplatin (for 3 days) to produce ototoxicity. After 1 week, ototoxicity was confirmed by testing of distortion-product otoacoustic emissions. Then, the rats were randomized to the following: (1) no treatment (control group), (2) ozone by rectal insufflation or (3) “ozone by rectal insufflation + intratympanic ozone administration”. Rectal and intratympanic insufflation were 2.3–3 mL of O_3_/O_2_ gas at concentration of 60 µg/mL. Ozone treatment was 1/day for 7 days. Rats were sacrificed after the 7th day. Compared with the control group, rats from both ozone groups showed statistical significance (*p* < 0.05): (1) better results in testing of distortion-product otoacoustic emissions (this is: partial recovery of audition), and (2) lower-outer hair cell damage in the histopathological examination score analysis of the inner ears. There were no differences observed between ozone groups. Therefore, it was concluded that rectal insufflation of ozone was effective in the treatment of cell damage in cisplatin-induced ototoxicity, and that the intratympanic administration of ozone had no additional advantage over the rectal administration. This study did not evaluate oxidative stress parameters.

Overall, the experimental models described above show that treatment with cisplatin was associated with a decrease in antioxidants, increase in free radicals and functional (creatinine) and histopathological damage in the kidneys and ears. However, the addition of ozone to the treatment was able to decrease all these alterations. These findings suggest a potential clinical benefit in the treatment and prevention of cisplatin-induced ototoxicity and nephrotoxicity, which are dose-limiting.

### 4.2. Ozone in Methotrexate-Induced Toxicity

In 2009, Kesik et al. [53] described the effect of ozone preconditioning to prevent abdominal injury by MTX, with assessment in liver, kidney and intestinal tissues. Ozone administration (total dose of 0.72 mg/kg) was by intraperitoneal route: one session/day for 15 consecutive days before the day of intraperitoneal MTX injection. Rats were sacrificed 5 days after MTX injection. They were evaluated in three groups: sham, MTX and MTX + ozone. Differences in free radicals and antioxidants among study groups were statistically significant and similar in all tissues: 1) Compared with sham, the MTX group showed an increase in MDA and decrease in SOD and GSH-Px compared with MTX alone; MTX + ozone showed decreased MDA and increased SOD and GSH-Px. Patterns of change were similar to those described in Figure 2. However, in this study, the histopathological scores for assessment of tissue damage were only statistically significant in ileum, which showed a lower damage score in the MTX + ozone group vs. MTX alone, that is: at histopathological level, the addition of ozone ameliorated intestinal damage at 5 days after MTX administration [53].

In 2015, Aslaner et al. published two articles with a similar methodology to evaluate the effect of “ozone preconditioning + ozone treatment” in MTX-induced nephrotoxicity [54] and hepatotoxicity [55]. The length of studies was 21 days. All groups received 5 mL of intraperitoneal administration of physiological saline (control and MTX groups) or O_3_/O_2_ (MTX + ozone groups). MTX and MTX + ozone groups received a single intraperitoneal administration of MTX at the 16^th^ day. Additionally, the MTX + ozone groups received O_3_/O_2_ (at 25 µg/mL) intraperitoneally, one session/day for 15 consecutive days before the MTX injection and five additional days after the MTX injection. Rats were sacrificed at the 21st day of the study. Compared with control groups, the MTX groups showed a significant increase in serum levels of ALT, ST, TNF-α and IL-1β and tissue levels of MDA and myeloperoxidase (MPO), as well as a significant decrease in tissue levels of GSH. However, compared with MTX alone, the MTX + ozone groups showed significantly lower serum levels of ALT, ST, TNF-α and IL-1β and tissue levels of MDA and MPO, as well as significantly higher tissue levels of GSH [54,55]. Compared with the MTX groups, the MTX + ozone groups showed a lower histopathological damage score, with statistically significant differences in kidney tissue. Patterns of change in MDA and GSH levels were similar to those described in Figure 2.

**Figure 3:**
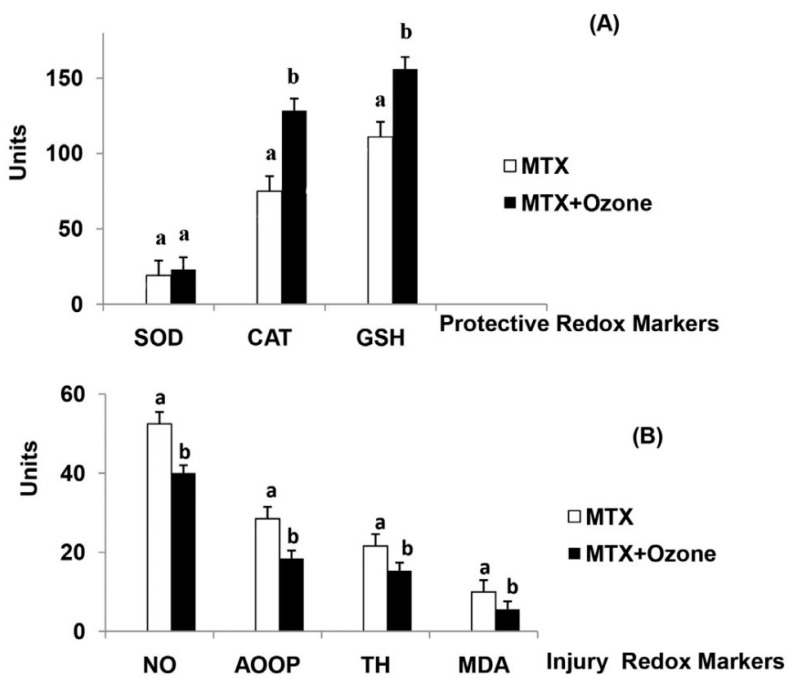
The redox status of patients with rheumatoid arthritis in (a): Methotrexate (MTX) and (b): “MTX + ozone” groups at the end of the study. (**A**) Protective redox markers, (**B**) Injury redox markers. The units of each marker are: SOD (superoxide dismutase, U/mL/min) and CAT (catalase, U/L/min) activities, GSH (reduced glutathione, µM), NO (nitric oxide, µM), AOPP (advanced oxidation protein products, µM), TH (total hydroperoxides, µM), MDA (malondialdehyde, µM). Data represent the mean ± S.E.M. of each group. Data analysis for each group was made by t-test. All differences between MTX vs. MTX + ozone groups were statistically significant, *p* < 0.05. From Ref. [56], with permission.

In 2016, Leon Fernandez et al. [56], described the results of a randomized controlled trial (RCT) using MTX without/with concurrent ozone therapy in patients with rheumatoid arthritis. Sixty patients were randomized into two groups to: (1) standard treatment (MTX group), with MTX (12.5 mg intramuscular) 1/week + Ibuprofen + folic acid; or (2) standard treatment + ozone (MTX + ozone group), with 20 rectal insufflations, 1/day, 5 days/week for 4 weeks. The O_3_/O_2_ concentration and volume were progressively increased, in order to enhance the adaptive response: from 25 µg/mL for 100 mL the 1st week to 40 µg/mL for 200 mL the 4th week. Patients in the MTX group only received standard treatment. Patients in the MTZ + ozone group received the same standard treatment + ozone by 20 rectal insufflation, 1/day, 5 days/week for 4 weeks. The O_3_/O_2_ concentration and volume were progressively increased, in order to enhance the adaptive response: from 25 µg/mL for 100 mL the 1st week to 40 µg/mL for 200 mL the 4th week. Clinical parameters and biochemical markers of oxidative stress were evaluated before and after the treatment. The MTX group showed no differences in disease activity score or health assessment questionnaire-disability index, whereas the MTX + ozone group showed a significant and clinically relevant improvement in both parameters, as well as a more remarkable decrease in pain intensity, according to the visual analog scale (VAS). Compared with patients treated in the MTX group, at the end of the study, patients treated with concurrent ozone therapy showed significantly higher levels of antioxidants (SOD, CAT, GSH) and lower levels of oxidative stress markers such as advanced oxidation protein products (AOPP), nitric oxide (NO), total hydroperoxides (TH) and malondialdehyde (Figure 3).

Overall, the works described above show that treatment with MTX was associated with a decrease in antioxidants, increase in free radicals and histopathological damage in kidney liver, and intestinal tissues. However, the addition of ozone to the treatment was able to decrease these alterations. These findings augur well for a potential clinical benefit of ozone in the treatment and prevention of MTX-induced toxicity in these issues, and they are further supported by the results in the only clinical trial published to date [57].

### 4.3. Ozone in Doxorubicin-Induced Toxicity

In 2004, Calunga et al. [58] described an experimental model of glomerulonephritis with a single DOX administration. After 10 weeks, rats were treated with O_3_/O_2_ rectal insufflation: one session/day for 15 days, at different concentrations. In this study, lower O_3_/O_2_ concentrations (15 µg/mL) showed better results than moderated concentrations (20 and 30 µg/mL) against the alterations induced by DOX on systolic arterial pressure, diuresis and proteinuria. However, this study did not evaluate the effect on oxidative stress or antioxidants. 

In 2014, Delgado-Roche et al. [57] described that ozone preconditioning could prevent DOX-induced cardiotoxicity. Rats were assigned to four groups: (1) control (without DOX), (2) DOX alone, (3) DOX + oxygen, and (4) DOX + ozone. Intraperitoneal DOX was administered twice a week for 50 days. The O_3_/O_2_ administration was by rectal insufflation, at a volume of 6 mL, and concentrations of 50 µg/mL in the DOX + ozone group and 0 µg/mL (only oxygen) in the DOX + oxygen group. In both O_3_/O_2_ groups, 20 sessions, 1/day, were administered before the commencement of DOX injection. Rats were sacrificed after 50 days. There were no significant differences between the DOX group and DOX + oxygen group. Compared with the control group, both showed: (1) a decrease in antioxidants (CAT and SOD) and (2) an increase in free radicals (MDA, AOPP) and pro-brain natriuretic peptide (pro-BNP) as a marker of cardiac damage. However, the DOX + ozone group showed levels of pro-BNP, free radicals and antioxidants that were significantly closer to those of the control group. Patterns of change in levels of pro-BNP, free radicals and antioxidants were similar to those described in Figure 2. Additionally, histopathological analysis of the DOX group showed significant damage in heart tissue (subendocardial loss of muscular fibres, mild edema, and necrosis), whereas the DOX + ozone group only showed minor damage [57]. 

**Figure 4:**
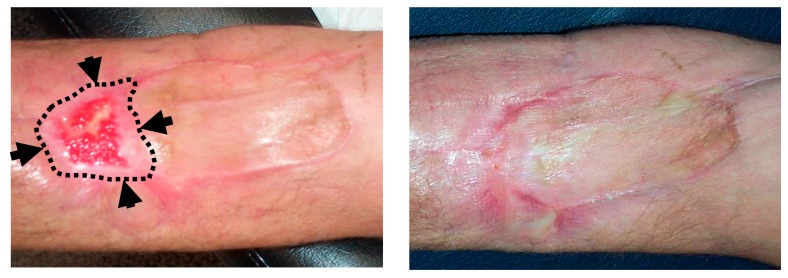
Topical ozone treatment in a patient with skin necrosis after Doxorubicin extravasation. A 61-year old patient under treatment for a stage IIIA multiple myeloma suffered a skin necrosis secondary to Doxorubicin (DOX) extravasation in the left elbow flexure. Because adverse evolution with conservative management, a muscle flap with a cutaneous graft was required (by the Department of Plastic Surgery). A second surgery was planned because of a loss of tissue in the distal area of the graft. (**Left**): Picture at the 9th session of local ozone therapy (wound size 25 × 15 mm). Black arrows and dotted lines show the limits of the wound at the commencement of ozone therapy (wound size 60 × 30 mm). (**Right**): Picture at the end of local ozone therapy, after 20 sessions. The planned second graft was avoided.

In 2016, Kesik et al. [59], described the effect of topical ozone application (ozonated olive oil) in the management of DOX-induced skin necrosis. This study assessed several topical treatments in an experimental model of skin necrosis induced by intradermal injection of Doxorubicin. The most relevant groups in this study were: (1) control group (DOX without further treatment), (2) DOX + dimethyl sulfoxide (DMSO), and (3) DOX + ozonated olive oil. It was expected the maximum skin necrosis occurred on day 14 after injection, so this was when analysis was carried out. Biopsies from the necrotic areas at 14 days did not show significant differences in tissue levels of MDA, IL1β, SOD or GSH-Px. However, compared with the control group, TNFα was significantly lower in DMS and ozonated olive oil groups, with no statistically significant differences observed between the last two groups. The ozonated olive oil group was the only one that showed a statistically significant decrease in ulcer size and in percentage of change (decrease) in the histopathologic ulcer score. DMSO is an antioxidant agent usually used in the management of DOX-induced extravasation injury. In this study, the authors demonstrated that topical use of ozonated olive oil improved this damage at least as well as DMSO [59]. Figure 4 shows a related clinical experience in our institution during the management of a patient with skin necrosis secondary to Doxorubicin extravasation. 

In 2017, Salem et al. [60] evaluated the cytoprotective effects of ozone (and rutin and their combination) on DOX-induced testicular toxicity. Intraperitoneal DOX was administered 3 times/week for 2 weeks since the commencement time-point. Since the same commencement time-point, all groups received rectal gas insufflation (5 mL): one session/day, 5 days/week for 3 weeks. Placebo and doxorubicin groups received insufflations with O_2_ only. In the ozone group, the gas insufflation was at O_3_/O_2_ concentrations of 25 µg/mL the 1st week, and 50 µg/mL the 2nd and the 3rd weeks. The study was terminated 21 days after treatment began. When compared to placebo, the DOX group showed a significant decrease in sperm count, motility and viability, and a significant increase in abnormal morphology. All these alterations were significantly lower in the group with DOX + ozone. In serum, DOX showed a significant decrease in testosterone levels and significant increases in luteinising hormone (LH) and follicle-stimulating hormone (FSH), whereas in the DOX + ozone group, these alterations were significantly lower. In testicular tissue, the DOX group showed a significant and relevant increase in g-glutamyltransferase (GGT), alkaline phosphatase (ALP), acid phosphatase, C-reactive protein (CRP), brain monocyte chemotactic protein-1 (MCP-1), malondialdehyde (MDA) and nitric oxide (NO), whereas all these values in the DOX + ozone group were significantly lower and closer to those of the placebo group. On the other hand, total antioxidant capacity (TAC) was significantly decreased in the DOX group, whereas in the DOX + ozone, there was a lower decrease and levels were closer to the placebo group. Patterns of change in MDA and antioxidant capacity were similar to those described in Figure 2. 

Finally, the recent work of Kamble et al. in 2018 merits mentioning. Using a different therapy (asiatic acid instead of ozone), they described that the activation of Nrf2 (as it is also induced by O_3_/O_2_ [7,27,37,38,51]) and the further enhancing of antioxidant systems can ameliorate DOX-induced toxicity in the heart, liver and kidneys [61].

Overall, the experimental models described above show that treatment with DOX was associated with a decrease in antioxidants, increase in free radicals and in functional and histopathological damage in the kidneys, heart, skin, and testicles. However, the addition of ozone to the treatment was able to ameliorate these alterations. These findings suggest a potential clinical benefit in the treatment and prevention of DOX-induced toxicity, and they are particularly relevant in DOX-induced cardiac-toxicity, which is dose-limiting.

### 4.4. Ozone in Bleomycin-Induced Toxicity

In 2015, Santana-Rodríguez et al. [62], showed preliminary results from an experimental model of Bleomycin-induced lung fibrosis. Twenty one Sprague-Dawley rats were randomized into four groups: (1) control, without intervention; (2) sham, with intratracheal administration of 500 μL saline; (3) BLM, with intratracheal administration of BLM; (4) BLM + ozone, treated as BLM group + O_3_/O_2_ rectal insufflation (20 mL/kg) before and after BLM administration. Administration of O_3_/O_2_ pre-BLM was 1/day for 15 days at increasing concentrations from 20 μg/mL to 50 μg/mL. After BLM administration, O_3_/O_2_ was administered at 50 μg/mL 3 times/week until sacrifice. Rats were sacrificed at 28 days after intratracheal administration of saline alone or with BLM. Lung fibrosis was assessed by the Ashcroft scale in a blinded histopathological analysis. Rats treated with BLM (with and without ozone) showed a significant and marked increase in lung fibrosis score. However, the fibrosis score was significantly lower in the BLM + ozone group in comparison with the BLM-alone group. Unfortunately, the levels of free radicals and antioxidants were not evaluated in this study [62]. 

### 4.5. Other Related Studies

There are few clinical works about the clinical effects of ozone in the management of CIT. They did not describe administered chemotherapy (or it was in a multidrug scheme) nor did they evaluate oxidative stress parameters. However, we consider that these are most relevant.

In 2008, a randomized study in children with chemo-induced mucositis showed that topical ozonated sunflower (Oleozon^®^, Centro Nacional de Investigaciones Científicas, La Habana, Cuba) leads to higher and faster mucositis recovery than conventional treatment with “Chlorhexidine + Nystatin” [63].

Borrelli, in 2012 [64], showed results from an RCT of 40 patients with advanced non-small lung cancer treated with (not specified) standard chemotherapy (control group) or standard chemotherapy and ozone (and viscum album injection). A concentration of O_3_/O_2_ of 30 µg/mL was administered by autohemotherapy once per week for 12 weeks. Compared with the control group, patients in the “chemotherapy and ozone group” showed a significant improvement in the Quality of Life Questionnaire QLQ-C30), lower ROS and higher biological antioxidant potential plasma values than baseline values.

Finally, a related topic to mention could be the chemotherapy-induced peripheral neuropathy (CIPN), which can happen in more than half of patients treated with platin compounds, taxanes, vincristine or bortezomib [65], and it can lead to dose-reduction or even interruption of chemotherapy. Once more, among the mechanisms associated to CIPN, the following have been described: (1) apoptosis induced by ROS and oxidative stress, (2) decrease in antioxidants as vitamin, E.; and (3) increase of proinflammatory cytokines (IL-1, IL-6, IL-8, TNFa) [65,66,67]. Different treatments have been evaluated, including several approaches with antioxidants: acetylcysteine, amifostine, glutathione, retinoic acid, or vitamin E. However, until now, preventive or therapeutic approaches are limited in number and efficacy [68,69]. In these clinical conditions, when non-proved or limited therapeutic options exist, some experts consider it reasonable to use treatment based on its mechanisms of action or its effects in related syndromes [69]. In this way, based on its mechanism of action and our clinical experience with ozone in neuropathic pain secondary to cancer treatments (*Personal Communication* [70]), a double-blinded RCT with ozone therapy in refractory peripheral neuropathy induced by chemotherapy is ongoing, which will include an extensive assessment of oxidative stress and proinflammatory parameters (EudraCT: 2019-000821-37).

## 5. Discussion and Prospects

Overall, in the experimental models described above, the administration of cisplatin, doxorubicin or methotrexate was associated with increased serum levels of tissue-damage markers (creatinine in renal injury, pro-BNP in cardiac injury) and increased tissue levels of free radicals (lipid peroxidation markers—TBARS, MDA). At the same time, these drugs decreased tissue levels of antioxidants (GSH, SOD, catalase, GSH-Px). When assessed, the addition of O_2_ (O_3_/O_2_ = 0 µg/mL) to rats treated with these chemotherapy drugs did not show relevant changes in comparison with chemotherapy alone, that is: the addition of systemic O_2_ did not induce a decrease of free radicals and did not increase antioxidant levels.

However, when the administration of cisplatin, doxorubicin or methotrexate in rats was associated with O_3_ preconditioning or O_3_ treatment at appropriate concentrations, the oxidative stress parameters were closer to the those from the control group, that is: (1) lower increase in serum levels of tissue-damage markers (creatinine in renal injury, pro-BNP in cardiac injury), (2) lower increase in tissue levels of free radicals (lipid peroxidation markers—TBARS, MDA), and lower decrease in the tissue levels of antioxidants (GSH, SOD, catalase, GSH-Px), 3) decreased damage in histopathologic analysis. Considering these effects, we know that oxidative preconditioning can induce an effect also described for other phenomena such as exercise or ischemic, thermal and chemical preconditioning. A common feature of all of these processes is that a repeated and “moderate-controlled” stress is able to protect against a prolonged and severe stress [44].

The results described with ozone in the experimental models detailed in this review augur well for a potential clinical benefit, and they are further supported by increased survival in the experimental model of doxorubicin plus ozone [57] or the results in the clinical trial of patients with arthritis treated with methotrexate with/without ozone therapy [56].

As with chemotherapy, chronic radiation-induced toxicity is also mediated by a local perpetuation of the ischemic process, proinflammatory and prooxidative status. Some experimental models have described the potential role of ozone therapy to protect/diminish toxicity at lung [71], liver or intestinal [72] levels. Unfortunately, once more, there are few related clinical studies, with the most remarkable being those regarding the use of ozone therapy during radiotherapy of prostate cancer to decrease local toxicity [73] or after radiotherapy to treat pelvic radiation-induced toxicity [70,74,75].

It is necessary to highlight that, as is usual in medicine, higher concentrations are not always better, as demonstrated by Borrego et al. [44], showing that cisplatin plus O_3_/O_2_ preconditioning at higher concentrations (50 and 70 µg/mL) showed histopathological changes that were quite similar to those present with cisplatin alone and disappointing results in biochemical parameters. Furthermore, the results were worse than those obtained with moderate concentrations (20 or 30 µg/mL) in rectal insufflations. That is to say, very high O_3_/O_2_ concentrations can induce free radical levels that are too high and exceed adaptive capacity, leading to worse results or even deleterious effects.

In the same way, the effects of 4-HNE, Nrf2 and NF-κB induced by ozone depend on its concentration and cell type or tissue. These three pathways interact in the redox processes and can show dual actions. If they lead to increased oxidative stress, they can induce initiation, promotion or progression of tumor cells, as well as treatment-induced toxicity; although an increase of oxidative stress is the foundation of chemotherapy and radiotherapy. On the other hand, an increase in antioxidants could be related with a lower risk of tumor initiation and treatment-induced toxicity; although, it could potentially protect cancer cells from cancer treatments. Therefore, their effects in cancer pathology and their potential modulation in cancer treatment are complex and not completely known [2,76,77].

At physiological (very low) doses, 4-HNE stimulates activity of the Nrf2 pathway as well as proliferation, differentiation, and apoptosis. However, low concentrations could protect cancer cells against further damage [2,77]. In opposition, studies have been described where 4-HNE correlates with tumor malignancy in astrocytomas and breast or liver carcinomas [2]; although, high levels of 4-HNE under oxidative stress conditions have been described to predispose cancer cells to apoptosis and enhance results of radio-chemo therapy in lung carcinomas [77,78]. The role of NF-κB and Nrf2 and their modulation in cancer pathology is also not clear. Coincident with the molecular cloning of NF-κB/RelA and identification of its kinship to the v-Rel oncogene, it was anticipated that NF-κB itself would be involved in cancer development. Oncogenic activating mutations in NF-κB genes are rare and have been identified only in some lymphoid malignancies, while most NF-κB activating mutations in lymphoid malignancies occur in upstream signaling components that feed into NF-κB. NF-κB activation is also prevalent in carcinomas, in which NF-κB activation is mainly driven by inflammatory cytokines within the tumor microenvironment. Importantly, however, in all malignancies, NF-κB acts in a cell-type-specific manner: activating within cancer cells genes involved in survival, proliferation, angiogenesis, expansion, and metastasis, as well as the enhancement of inflammation-promoting genes in the tumor microenvironment. Yet, the complex biological functions of NF-κB have made targeting it therapeutically a challenge [79,80]. Moreover, Nrf2 has also shown a dual action that can enhance resistance to cancer treatment as well as inhibit cancer initiation and development [76]. Nrf2 increase has been associated with malignant transformation and progression in colorectal carcinoma [81], limited the success of temozolomide and is implied to play a role in the drug resistance mechanism [82] in gastric cancer. Nrf2 expression is positively correlated with invasive gastric cancer, suggesting its utility as a predictive index for unfavorable prognosis [83]. However, controlled, oscillating activation of Nrf2 has also been related to the prevention of cancer initiation and development [76,84]. In conclusion, it seems that modification of the balance of Nrf2 or NF-κB is involved in regulation of cancer initiation/progression and the drug resistance mechanism. As consequences, approaches that reestablish the equilibrium Nrf2/NF-κB should provide a potential benefit in oncology. 

As commented for 4-HNE, NF-κB, and Nrf2, the use of “high-dose antioxidants” during chemotherapy to prevent toxicity is also controversial, because of the potential protective effect on tumor cells and prognostic impairment [85,86]. However, ozone does not lead to a high increase of one isolated substance or antioxidant. At appropriated concentrations, ozone will induce an adaptive response with an “overall potentiation” of the “endogenous antioxidant mechanisms”, which are usually decreased in most tumor cells. 

There is a rational support for a potential enhancing effect of the “chemotherapy + ozone” combination as we have described in a recent review, which merits further research [87]. However, this potential and controversial combination during cancer treatment should not be hugely relevant for patients in the following situations: 

*(1) Current or potential CIT leads to contraindication or dose-reduction chemotherapy.* In some clinical conditions, the addition of ozone during chemotherapy in order to prevent/diminish CIT could open two additional interesting treatment-windows to explore: (a) to avoid/diminish chemotherapy dose-reduction when some kind of CIT is present, and (b) the potential possibility for exploring chemotherapy administration in current clinical contraindications (e.g., renal failure).

*(2) Tumor cells are not present, e.g., in the treatment of CIT after cancer treatment*. Based on the demonstrated modulation of oxidative stress by ozone, the complementary use of ozone as palliative or compassionate treatment for CIT could be supported when an effective or demonstrated treatment does not exist or does not work, as suggested by experts [69]. In this way, an RCT in refractory peripheral neuropathy induced by chemotherapy is ongoing (EudraCT: 2019-000821-37), with planned analysis of inflammatory and oxidative stress markers.

*(3) Chemotherapy is used in the management of no-cancer disease*. This is supported by the RCT in rheumatoid arthritis, where the addition of ozone therapy to MTX treatment improved the biochemical and clinical results [56]. 

There is no doubt that all the above-mentioned issues merit further research and RCT.

## 6. Conclusions

The relationship between free radicals and ROS vs. antioxidants is a complex balance that depends on their concentrations and cell/tissue type of action, and with a Janus effect—both sides of the balance can lead to beneficial or harmful effects. Increased oxidative stress is associated with cancer and CIT, although a further increase of oxidative stress in cancer cells is key in chemotherapy and radiotherapy actions. On the other hand, high antioxidant levels could be useful in the management of CIT, although we must be careful with the potential protective effect on cancer cells. Ozone therapy, by an initial “soft and controlled” oxidative stress induces an adaptive response of the tissues with a final increase of the “overall-endogenous antioxidant systems”, which have been associated with protective and therapeutic effects in CIT in several experimental models and an RCT. The potential benefit of ozone in these clinical conditions merits further research. 

## Abbreviations

4-HNE: 4-hydroxynonenal; AOPP: advanced oxidation protein products; BLM: Bleomycin; CAT: catalase; CDDP: Cisplatin; CIT: chemotherapy -induced toxicity; CSGF: colony-stimulating growth factors; CT: chemotherapy (CT); DOX: Doxorubicin; G6PDH: glucose-6-phosphate dehydrogenase; GSH: reduced glutathione; GSH-Px: glutathione peroxidase; H_2_O_2_: hydrogen peroxide; HO-1: heme oxygenase-1; IL: interleukin; MDA: malondialdehyde; MPO: myeloperoxidase; MTX: Methotrexate; MPO: myeloperoxidase; NO: nitric oxide; NF-κB: nuclear factor kappa B; Nrf2: nuclear factor erythroid 2-related factor; O_2_: oxygen; O_2_.-: superoxide anion; O_3_: ozone; OH•: hydroxyl radical; Prxs: peroxiredoxins; RCT: randomized controlled trial; ROS: reactive oxygen species; SOD superoxide dismutase; TAC: total antioxidant capacity; TBARS: thiobarbituric acid-reactive substances; TH: total hydroperoxides; TNF-α: tumor necrosis factor-alpha
